# Dalbavancin for infective endocarditis: A systematic review of effectiveness, safety, and dosing

**DOI:** 10.1007/s10096-026-05434-3

**Published:** 2026-02-10

**Authors:** Anastasios E Chaldoupis, Vasilios Petrakis, Petros Ioannou, Konstantina A Tsante, Deny Tsakri, Aglaia Domouchtsidou, Stella Baliou, Marianna Vlachaki, Alexandra Mpakosi, Sotirios P Fortis, George Samonis, Rozeta Sokou, Stefanos Bonovas, Gerasimos Siasos, Andreas G Tsantes

**Affiliations:** 1 Department of Cardiology, Medical School, Sotiria Chest Disease Hospital, National and Kapodistrian University of Athens, Athens, 11527 Greece; 2 Department of Medicine, Infectious Diseases Unit, University General Hospital ofAlexandroupolis, Evros, Greece; 3 School of Medicine, University of Crete, Heraklion, 71003 Greece; 4 Department of Biomedical Sciences, University of West Attica, Athens, 12243 Greece; 5 Department of Microbiology, Medical School, National and Kapodistrian University of Athens, Athens, 11527 Greece; 6 Microbiology Department, Saint Savvas Oncology Hospital, Athens, 11522 Greece; 7 Laboratory of Toxicology, School of Medicine, University of Crete, Heraklion, 71003 Greece; 8 Department of Microbiology, General Hospital of Nikaia Agios Panteleimon, Piraeus, 18454 Greece; 9 Department of Immunology, General Hospital of Nikaia Agios Panteleimon, Piraeus, 18454 Greece; 10 Laboratory of Reliability and Quality Control in Laboratory Hematology (HemQcR), Department of Biomedical Sciences, School of Health & Caring Sciences, University of West Attica (UniWA), Egaleo, Greece; 11 First Department of Medical Oncology, Metropolitan Hospital of Neon Faliron, Athens, 18547 Greece; 12 Neonatal Department, National and Kapodistrian University of Athens, Aretaieio Hospital, Athens, 11528 Greece; 13 Department of Biomedical Sciences, Humanitas University, Pieve Emanuele, Milan, 20072 Italy; 14 IRCCS Humanitas Research Hospital, Rozzano, Milan, 20089 Italy; 15 Laboratory of Haematology and Blood Bank Unit, School of Medicine, Attiko Hospital, National and Kapodistrian University of Athens, Athens, 12462 Greece

**Keywords:** Systematic review, Dalbavancin, Infective endocarditis, Antimicrobial therapy, Treatment outcomes

## Abstract

**Purpose:**

Infective endocarditis (IE) requires prolonged intravenous antibiotic therapy, leading to extended hospitalization and increased morbidity. Dalbavancin, a long-acting antibiotic with excellent tissue penetration and a favorable safety profile, can be a promising alternative. This systematic review aimed to investigate the current literature regarding the use of dalbavancin for infective endocarditis.

**Methods:**

A systematic search of PubMed and Scopus was conducted according to PRISMA guidelines. Eligible studies included adult patients (≥ 18 years) with IE diagnosed by the modified Duke criteria, treated with dalbavancin as monotherapy or sequential/consolidation therapy. Data extraction included demographics, type of IE, causative pathogen, dosing regimen, surgical intervention, adverse events, and outcomes. Descriptive analyses were performed.

**Results:**

Thirty-eight studies including 565 patients were analyzed. The overall cure rate was 88.0%, with a similar success rate across native valve (90.1%), prosthetic valve (90.0%), and cardiac device-related IE (86.0%). *Staphylococcus aureus*, Coagulase-negative staphylococci (CNS), *Streptococcus spp*., and *Enterococcus faecalis* were the most common pathogens. Cure rates were comparable among most pathogens, though a lower cure rate was evident in infections due to *Enterococcus faecalis* compared to *Streptococcus spp.* (80.7% vs. 96.6%, *p* = 0.008). An effective antimicrobial exposure of 2 weeks had similar success rate compared to a longer period of effective antimicrobial exposure (*p* = 0.31). Adverse events were rare (2.9%), with mild rash being the most common.

**Conclusions:**

Dalbavancin demonstrates high cure rates and excellent tolerability in IE, and could offer an attractive alternative to conventional prolonged intravenous therapy. Further randomized controlled trials are warranted to define standardized protocols.

**Supplementary Information:**

The online version contains supplementary material available at 10.1007/s10096-026-05434-3.

## Introduction

Infective endocarditis (IE) is a potentially life-threatening infection associated with devastating complications such as perivalvular abscess and pulmonary embolism, while it often requires surgical intervention in order to achieve adequate source control [1]. Given the intricacy of this disease, a prolonged period (4–6 weeks) of intravenous antibiotic therapy is recommended, resulting in long-term hospitalization, associated not only with increased morbidity, but also with a substantial financial burden [1, 2]. Therefore, recent research is focused on shorter IV antibiotic therapy and earlier onset of oral treatment [3, 4]. However, there is a lack of evidence regarding the effectiveness of oral treatments, especially in specific populations, such as in patients who inject drugs (PWID).

Dalbavancin is a semi-synthetic lipoglycopeptide derived from A40926, a glycopeptide similar to teicoplanin, but with a prolonged half-life due to its high protein-binding capacity [5]. Dalbavancin has a wide antibiotic spectrum including mainly Gram-positive bacteria, while the altered pharmacodynamics and pharmacokinetics of dalbavancin’s modified molecule make this antibiotic a favorable option [6]. Its prolonged half-life allows for extended dosing intervals of more than 1 week, while its excellent tissue penetration and ability to prevent biofilm formation make it an ideal antibiotic for deep-seated infections such as endocarditis, even though its main approved indication is for the treatment of skin and soft tissue infections [7, 8]. A great benefit from using dalbavancin in patients with endocarditis arises for people who inject intravenous drugs. This population often leaves hospital against medical advice, leading to relapses, while the peripherally inserted central catheter for consolidation therapy could serve as a means to inject substances [9]. Considering all the aforementioned points, the administration of a single dose dalbavancin on the day of discharge with or without subsequent doses, appears an ideal option for these patients.

In addition, only 40% of dalbavancin is excreted renally, while its metabolism does not involve cytochrome P-450, composing an excellent safety profile with minimal drug interactions and adverse events [6]. Therefore, adjustment for renal or hepatic impairment is not required, except for patients with creatinine clearance < 30 ml/min who are not under hemodialysis [7]. All the above characteristics provide a financial benefit on account of the earlier hospital discharge and the remission of high-cost extra-hospital facilities [10]. Therefore, despite the fact that dalbavancin has been currently approved only for acute bacterial skin and skin structure infections, the off-label use in patients with endocarditis is very ubiquitous in clinical practice [8, 11].

The purpose of this review is to investigate the current literature regarding the use of dalbavancin against different types of infective endocarditis and its causative pathogens, focusing on the various treatment protocols, the effectiveness, and the safety profile of this new antibiotic.

## Methods

### Search Protocol/Databases

In order to identify and evaluate studies relevant to the aim of this systematic review, a protocol was developed in accordance with the PRISMA (Preferred Reporting Items for Systematic Reviews and Meta-Analyses) guidelines, and the review protocol was registered in the PROSPERO database (CRD420251127789; registered 2025).

The aim of this study was to identify studies presenting the clinical experience with dalbavancin in cases of infective endocarditis, focusing on its effectiveness, safety, and treatment regimens used. The literature search was conducted between May 2025 and July 2025. Electronic searches were performed in the PubMed and Scopus databases for studies published from inception until July 2025, using a combination of keywords including: “new glycopeptides”, “lipoglycopeptides”, “dalbavancin” and “endocarditis”. The detailed search strategy is provided in Supplementary **(Table ****S1****)**. An initial screening of titles and abstracts was carried out to exclude clearly irrelevant studies. As a following step, full texts of the potentially eligible articles were then assessed thoroughly to determine whether they met the inclusion criteria of the study. The selection and extraction process were conducted by two independent reviewers (AC and AGT), while any disagreement was resolved by a third reviewer (SB). Screening of studies was performed using the Rayyan web application, which facilitated independent review and selection based on predefined inclusion and exclusion criteria. Additionally, reference lists of the selected articles were manually screened to identify further eligible studies.

## Inclusion and exclusion criteria

Only studies published in English were considered eligible. Studies with original data (Randomized Clinical Trials, (RCTs), observational studies, case reports, case series, cohort studies) reporting results of dalbavancin administration, either as monotherapy or part of a sequential/consolidation regimen for confirmed cases of infective endocarditis in adult population (≥ 18 years old) were searched. Infective endocarditis was diagnosed according to modified Duke criteria [12]. Review articles, meta-analyses, editorials, letters, and conference abstracts, while also studies involving mixed populations where data on the target group could not be separated were excluded. Moreover, studies including animal experiments, in vitro analyses, pharmacoeconomic evaluations, or reviews without original clinical data were also excluded.

## Data collection and extraction

Data was manually extracted from the included studies and organized into multiple structured Microsoft Excel datasheets. For each case or cohort, we recorded: demographics (gender and age), type of IE (Native valve endocarditis [NVE], prosthetic valve endocarditis [PVE], cardiac device-related IE [CDIE], or vascular graft infection [VGI]), microbiological profile (Pathogen isolated [e.g., MSSA, MRSA, Streptococcus spp., Enterococcus faecalis, etc.]), dosing regimen (type and number of dalbavancin doses administered), total dose administered (mg), duration of therapy (weeks), surgical intervention (whether patient underwent surgery), outcome (Cured, failure, or lost to follow-up). Cure was defined as remission of signs and symptoms, or as sterilization of blood cultures or as normalization of laboratory values.

### Data analysis

Considering the heterogeneity and the observational nature of most included studies, conducting a comprehensive and rigorous meta-analysis was deemed inappropriate and unlikely to yield meaningful results. However, we attempted to extract patient-level data where possible, in order to draw to the extent feasible clear conclusions, regarding the effectiveness of dalbavancin across different patient subgroups, based on endocarditis type, causative pathogens, treatment characteristics such as dose and duration and the presence or absence of surgical intervention. Descriptive statistics of the above parameters were performed. Statistical analysis was performed using Stata 19.5 (StataCorp, College Station, TX, USA) software. Comparisons of variables were performed using chi-square test, as appropriate. A p-value < 0.05 was considered statistically significant for all tests.

## Results

The electronic search identified a total of 652 articles in PubMed and Scopus databases, while after duplicate removal, 558 studies remained available for the initial screening. The titles and abstracts were screened, and 486 articles were removed because they were irrelevant to the topic of interest. The full text of the remaining 72 articles underwent a thorough review to evaluate whether they met the inclusion criteria. Following this full text review, 34 studies were excluded because either they were not reporting outcomes regarding treatment success/failure (*n* = 17), or because their population was included in a larger study (*n* = 3), or they were review articles (*n* = 14).

**Fig. 1:**
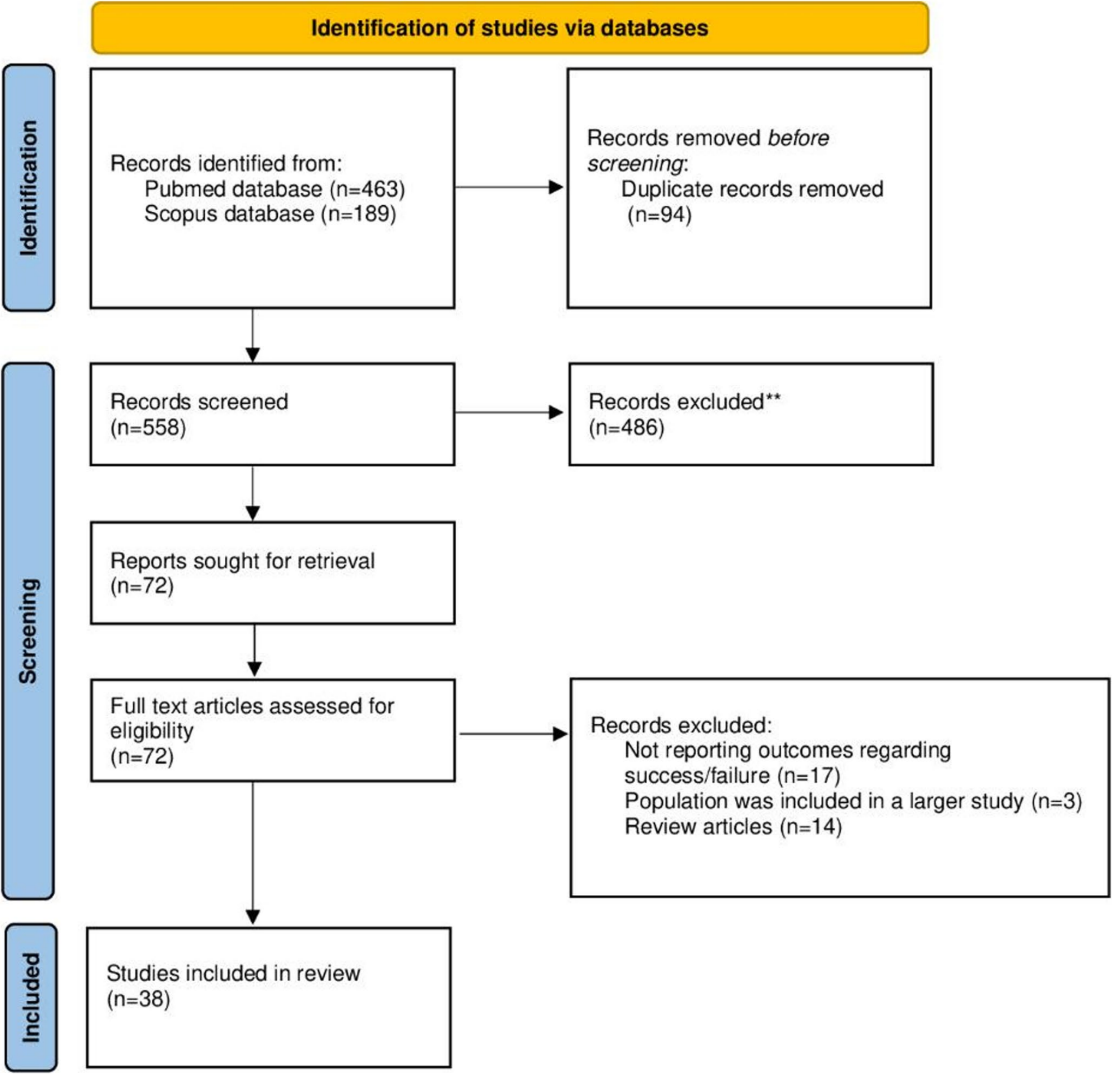
Diagram of the search and selection process (flow chart)

Finally, 38 studies were included in the study evaluating 565 patients who received dalbavancin for IE [13–50]. (Fig. 1). None of these studies was a randomized controlled trial (RCT), while there were 24 observational studies (1 prospective [50] and 24 retrospective [13, 19, 20, 22, 25–31, 33–35, 38–44, 46, 48, 49]), 6 case series [14, 24, 32, 36, 45, 47], and 7 case reports [15–18, 21, 23, 37]. Most studies were conducted in the United States [13, 16, 19, 20, 23, 24, 26, 30, 31, 40, 41, 45] (*n* = 12) and Spain [15, 17, 18, 28, 32–34, 43, 46, 48] (*n* = 11), with additional studies from Italy [14, 21, 35, 42] (*n* = 4), Austria [25, 37, 39] (*n* = 3), United Kingdom [29, 44, 47] (*n* = 3), France [27, 50] (*n* = 2), Canada [49] (*n* = 1), Germany [36] (*n* = 1) and Greece [22] (*n* = 1). Among the total cohort of 565 patients with reported outcomes, 497 (88.0%) patients achieved cure, while 68 (12.0%) patients failed therapy.

Dalbavancin was predominantly administered as sequential or consolidation therapy following an initial course of standard intravenous antibiotics. Only 6 patients received dalbavancin without any prior antibiotic therapy. Specifically, a single case was reported by Hakim et al., while 3 patients were reported in a cohort study by Tobudic et al., and 2 patients in cohort study by Morata et al. [16, 25, 46]. Due to the limited number of patients and incomplete outcome reporting for patients in whom dalbavancin was used as the sole antimicrobial treatment, subgroup analysis based on treatment strategy (sequential therapy versus monotherapy) was not feasible.

### Types and Microbiological profile of IE

The type of IE was reported for 464 patients in 27 studies [13–18, 21, 23–25, 28, 30–39, 44–48, 50]. Regarding the type of IE, Native Valve Endocarditis (NVE) was the most common type (*n* = 223, 48.1%), followed by Prosthetic Valve Endocarditis (PVE; *n* = 49, 41.1%) and Cardiac Device-Related Infective Endocarditis (CDIE; *n* = 50, 10.8%). The overall cure rate for NVE was 90.1%, while the cure rate of PVE was 90.0% and of CDRIE was 86.0%. The cure rates were similar among the different types of IE (NVE vs. PVE: *p* = 0.97, NVE vs. CDRIE: *p* = 0.39, CDIE vs. PVE: *p* = 0.41). Table 1 presents the distribution of outcomes by type of infective endocarditis (IE).

**Table 1:** Effectiveness of Dalbavancin based on type of infective endocarditis

Diagnosis	Total Cases	Cured (%)
Native Valve Endocarditis	223	201 (90.1)
Prosthetic Valve Endocarditis	191	172 (90.0)
Cardiac Device-Related Infective Endocarditis	50	43 (86.0)
Total	464	416 (89.6)

**Fig. 2:**
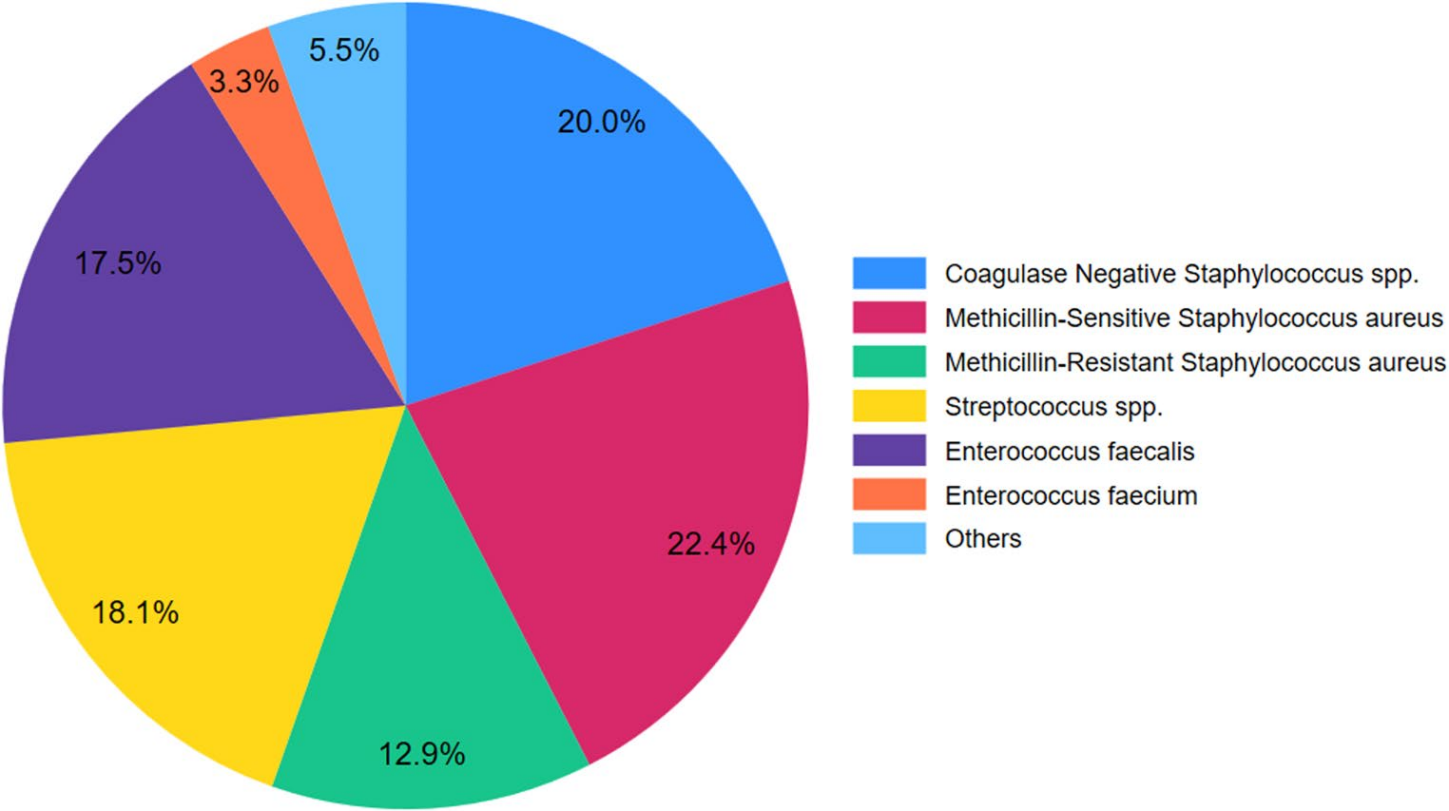
Microbiological profile of patients with IE

Isolated pathogens were reported for 325 patients in 25 studies [14, 18, 20, 21, 23–25, 28, 30–38, 43, 45–48]. The most common isolated pathogens in patients with infective endocarditis who received dalbavancin included *Staphyloccocus aureus* (*n* = 115, 35.3%), followed by coagulase-negative staphylococci (CNS) (*n* = 65, 20.0%), *Streptococcus spp.* (*n* = 59; 18.1%), *Enterococcus faecalis* (*n* = 57, 17.5%; Fig. 2). Among the patients with infections due to *Staphylococcus aureus*, methicillin-resistant *Staphylococcus aureus* (MRSA) was isolated in 42 (12.9%) patients, and methicillin-sensitive *Staphylococcus aureus* (MSSA) in 73 patients (22.4%). The cure rate of patients with infections due to MRSA (90.4%) was similar to that of patients with MSSA (90.4%, *p* = 0.99). Similarly, the cure rate of patients with infections due to CNS (89.2%) was similar to that of patients with MRSA (90.4%, *p* = 0.83), MSSA (90.4%, *p* = 0.81), Streptococcus species (96.6%, *p* = 0.11), and Enterococcus faecalis (80.7%, *p* = 0.18). The only difference between cure rates was observed between patients with infections due to Enterococcus faecalis and Streptococcus species, with a lower cure rate for those with Enterococcus faecalis (80.7% vs. 96.6%, *p* = 0.008) (Table 2).

**Table 2:** Microbiological profile

Microorganism	Total cases	Cured (%)
Coagulase Negative *Staphylococcus* species	65	58 (89.2)
Methicillin-Sensitive *Staphylococcus aureus*	73	66 (90.4)
Methicillin-Resistant *Staphylococcus aureus*	42	38 (90.4)
*Streptococcus* species	59	57 (96.6)
*Enterococcus faecalis*	57	46 (80.7)
*Enterococcus faecium*	11	11 (100.0)
Other *Enterococcus* species	3	3 (100.0)
Other *Staphylococcus* species	2	0 (0.0)
Other species	13	12 (92.3)
Total	325	291 (89.5)

## Dosing schemes of Dalbavancin

**Fig. 3:**
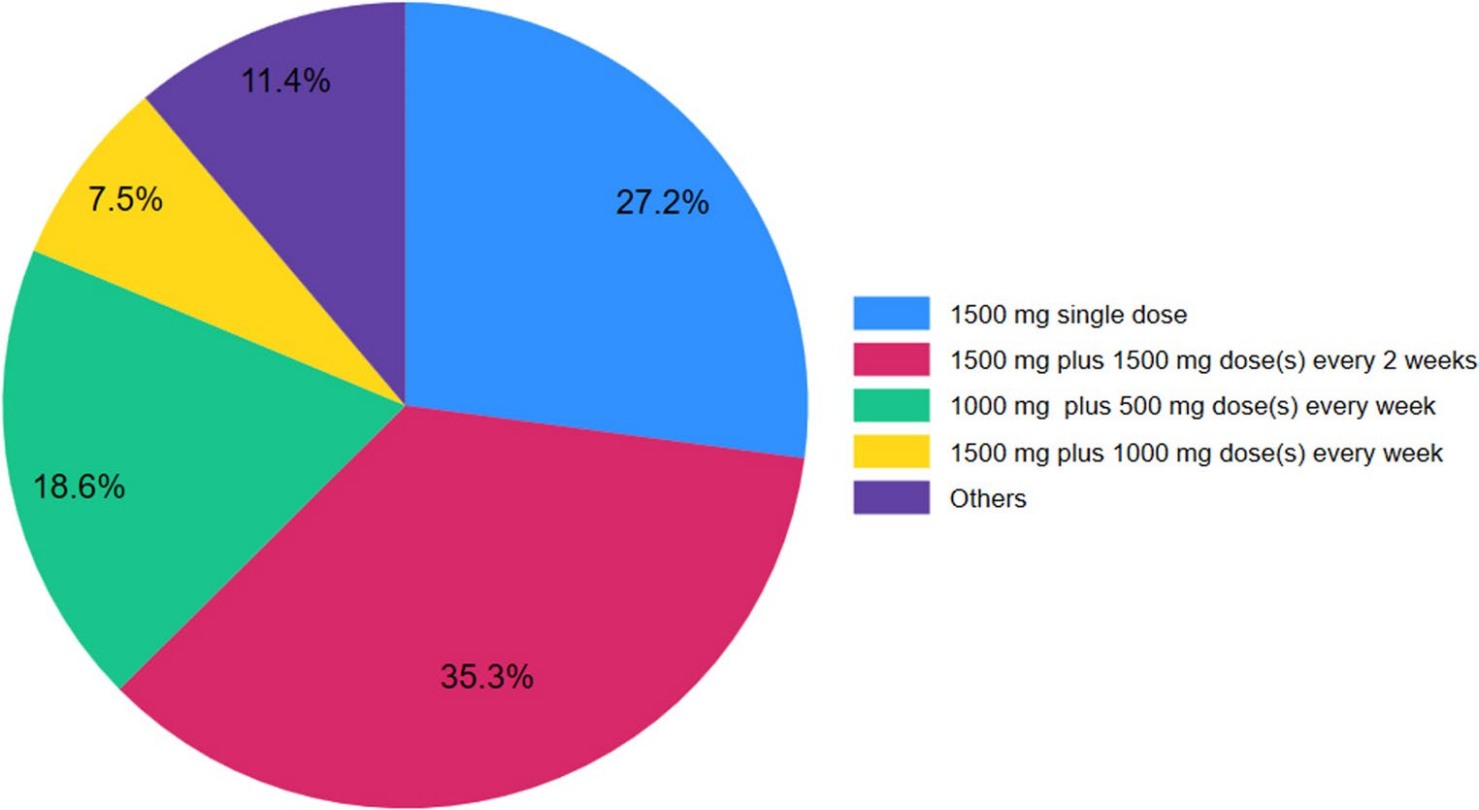
Dosing schemes of dalbavancin in the population

Overall, 11 different dosing schemes of dalbavancin for the treatment of IE were described for 359 patients in 24 studies [14–18, 20, 21, 23–25, 28, 30–35, 38–40, 46–49] (Table 3). A dosing scheme including an initial dose of 1500 mg plus dose(s) of 1500 mg administered at two-week interval was the most common scheme (*n* = 127, 35.3%), followed by a single dose scheme of 1500 mg (*n* = 98, 27.2%), and a scheme of 1000 mg as an initial dose plus dose(s) of 500 mg administered at a 1-week intervals (*n* = 67, 18.6%; Fig. 3). The success rate of the most common dosing scheme including an initial dose of 1500 mg plus dose(s) of 1500 mg administered at two-week intervals was higher than that of the single dose (1500 mg) scheme (94.4% vs. 83.6%, *p* = 0.008), but similar to that of the third most common dosing scheme including an initial dose of 1000 mg plus 500 mg dose(s) every week, which was 91.0% (*p* = 0.36). Moreover, the success rate of the single dose (1500 mg) scheme was similar to that of the scheme including 1000 mg as an initial dose plus dose(s) of 500 mg administered at a 1-week intervals (*p* = 0.17).

**Table 3:** Dosing schemes of Dalbavancin

Scheme	Total cases	Cured (%)
1500 mg single dose	98	82 (83.6)
1000 mg single dose	24	23 (95.8)
1500 mg initial dose plus 1500 mg dose(s) - (2 weeks interval)	127	120 (94.4)
1000 mg initial dose plus 1000 mg dose(s) - (2 weeks interval)	4	4 (100.0)
500 mg initial dose of plus another dose	3	1 (33.3)
1500 mg initial dose plus 1000 mg dose(s) every week	27	26 (96.2)
1500 mg initial dose plus 500 mg dose(s) every week	4	4 (100.0)
1000 mg initial dose plus 500 mg dose(s) every week	67	61 (91.0)
750 mg single dose	1	1 (100.0)
1000 mg initial dose plus 375 mg dose(s)	1	1 (100.0)
1500 mg initial dose plus 1500 mg plus 1000 mg every 2 weeks	3	1 (33.3)
Total	359	287 (90.2)

## Total dose, duration and surgical intervention

The total dose of dalbavancin was reported for 228 patients in 17 studies [14, 16, 17, 20, 23, 24, 28, 30, 32, 33, 35, 36, 39, 40, 46–48]. The total median dose of dalbavancin in the overall population was 1500 mg (Interquartile range [IQR]: 1500–3000). The total dose was similar between patients with successful treatment and those with failed treatment (1500 mg for both groups, *p* = 0.68).

The number of doses and total duration of dalbavancin treatment was reported for 329 patients, in 24 studies [14–18, 20, 21, 23–25, 28, 30, 32–37, 39, 40, 46–49]. Given the prolonged half-life and sustained therapeutic concentrations of dalbavancin, treatment duration was defined as the estimated period of effective antimicrobial exposure rather than the calendar interval between doses. Specifically, dosing regimens were translated into effective treatment durations based on pharmacokinetic data (e.g., a single 1500 mg dose corresponding to approximately two weeks of therapeutic exposure). Separately, the total number of dalbavancin doses administered was recorded as an independent variable. Overall, 123 (37.8%) patients received one dose of dalbavancin, 140 patients received two doses (42.5%), while 66 patients received more than two doses. The median duration of effective antimicrobial exposure with dalbavancin treatment was 3 weeks (IQR:2–4). The median duration of effective antimicrobial exposure for patients with successful outcome was 3 weeks (IQR: 2–4), while the median duration for those who failed treatment was also 3 weeks (IQR: 2–4). Patients with a period of effective antimicrobial exposure of 2 weeks had similar success rate compared to those with a longer effective antimicrobial exposure (89.2% vs. 92.4%, *p* = 0.31; Table 4).

Information regarding surgical intervention was reported for 310 patients, in 21 studies [14–18, 21, 23, 25, 28, 32–38, 44–48]. Surgical intervention was performed in 116 patients (37.4%). The treatment success rate in patients who underwent surgery was 88.7%, similar to the treatment success rate in patients who did not undergo surgery, which was 85.0% (*p* = 0.35; Table 4).

**Table 4:** Duration of treatment and surgical intervention in the population

Parameters	Total cases	Cured (%)	*p*-value
Duration of effective antimicrobial exposure 2 weeks >2 weeks	130 199	116 (89.2) 184 (92.4)	0.31
Surgical intervention Yes No	116 194	103 (88.7) 165 (85.0)	0.35

Presence of any adverse event was evaluated in 373 patients from 23 studies [13, 15–18, 20, 21, 23–25, 28, 30, 32–38, 44, 47–49]. Overall, 11 (2.9%) adverse events were reported in the population. The most common adverse events included rash after infusion (*n* = 5, 1.3%), followed by pseudomembranous colitis (*n* = 2, 0.5%), Moreover, 1 patient presented with impaired renal function (0.026%), 1 patient presented with fatigue (0.026%), 1 patient with elevated hepatic enzymes (0.026%), and 1 patient presented with thrombopenia and epistaxis (0.026%).

## Discussion

Dalbavancin is associated with several beneficial properties such as a prolonged half-life allowing for extended dosing intervals, excellent tissue penetration, and favorable safety profile. Based on published reports, the cure rate of dalbavancin for infective endocarditis ranges from 70% to 85%, depending on the patient population, pathogen resistance, and surgical intervention [51–54]. Many studies have also shown that dalbavancin is associated with major cost savings [26, 28, 38, 55, 56]. Although constituting an expensive drug, sparing from the earlier hospital discharge and the unnecessary high cost of outpatient parenteral antibiotic therapy results in considerable financial benefit. Moreover, the expected lower incidence of drug’s adverse events and catheter related complications such as infection or thrombosis, implies additional savings [57, 58]. This is the largest systematic review, consolidating evidence from 38 studies in literature, tracing out the administration of dalbavancin in cases of infective endocarditis. Based on the results of this study, the overall treatment outcome was highly favorable, with a cure rate of 88.0% in patients with infective endocarditis.

Although pharmacokinetics of dalbavancin is quite predictable, the therapeutic index may widely vary and optimal concentrations may not always be achieved, especially in patients with renal impairment, extreme body mass index, or prolonged suppressive therapy. In this context, therapeutic drug monitoring (TDM) is a very useful tool to ensure sustained therapeutic exposure and minimize the risk of subtherapeutic concentrations especially in cases of endovascular infections where prolonged and off-label use is required. Recent expert opinion panels have suggested that TDM may be considered in cases of long-term dalbavancin administration [7]. Unfortunately, consistent information regarding dalbavancin TDM in patients treated for infective endocarditis is lacking in the literature. Future studies evaluating the efficacy of dalbavancin in patients with infective endocarditis should also assess the clinical utility of TDM-guided dalbavancin therapy.

The most commonly reported pathogens included Coagulase-negative staphylococci (20.0%), MSSA (22.4%) and *Streptococcus spp*. (18.0%), while *Enterococcus faecalis* accounted for 17.5% of cases and MRSA for 12.9% of cases. These findings align with the expected microbiology of IE, highlighting dalbavancin’s utility against Gram-positive cocci [59]. The effectiveness of dalbavancin was similar for most pathogens, with the only difference being observed between the cure rates against *Enterococcus faecalis* and *Streptococcus spp*., indicating a lower cure rate in patients with *Enterococcus faecalis*. However, the small number of reported cases for certain pathogens does not allow for safe conclusion regarding the comparative effectiveness of dalbavancin. For example, the overall cure rate for infections due to *Enterococcus faecium*, a pathogen that possesses intrinsic resistance to several common antibiotics, was found to be 100%. However, this finding does not reflect the true virulence of this pathogen and probably the true effectiveness of dalbavancin against it, since only 11 cases of IE due to *Enterococcus faecium* were reported.

As reported, Enterococcus faecalis was associated with lower cure rates compared to streptococcal infections. Although dalbavancin exhibits an effective bactericidal activity against vancomycin-susceptible enterococci, it lacks activity against vancomycin-resistant enterococci (VRE). Oritavancin is another long-acting lipoglycopeptide that differs pharmacodynamically from dalbacancin, exhibiting in vitro activity against both vancomycin-susceptible enterococci and certain VRE phenotypes (VanA and VanB). Although clinical data on oritavancin use in infective endocarditis are sparse and largely limited to case reports and small case series, its broader enterococcal spectrum may represent a theoretical advantage in selected scenarios.

Regarding the type of IE, dalbavancin demonstrated consistent effectiveness across major IE types, with slightly higher failure rate in CDIE. To the best of our knowledge, this is the first study providing stratified cure rates based on the type of infective endocarditis, offering clinically relevant information that may contribute to more individualized therapeutic considerations in different patient populations. However, we have to note that the type of infective endocarditis was not specified in many cases, resulting in substantial missing data. This is true for even large multicenter cohort studies such as the EN-DALBACEN 2.0 study, that although they provide extensive data regarding the effectiveness of dalbavancin, they do not include any subgroup analysis based on the type of endocarditis [25, 28, 46].

Clinical failure of dalbavancin is associated with inadequate source control, which in many cases requires surgical intervention [14, 25, 31, 39]. Overall, 37.4% of the patients included in the review underwent surgical intervention. The role of surgery for adequate source control in infective endocarditis is pivotal. However, the association between surgical intervention and successful outcomes may be confounded by several factors. Although a higher cure rate is expected in patients who underwent successful surgery, there are cases where surgery fails to achieve complete source control, requiring consecutive surgeries. In such cases, a lower cure rate is anticipated. Moreover, surgical intervention may indicate a more severe disease, where a lower cure rate is also expected. Hidalgo-Tenorio et al. evaluated the effectiveness of dalbavancin as consolidation therapy in infective endocarditis due to *Enterococcus spp*. The authors reported that patients who were indicated and underwent surgery showed a higher clinical cure rate (100 vs. 75.8%; *p* = 0.005). Unfortunately, similar data regarding cure rates according to surgical management were missing in many studies.

Identifying the ideal duration and dosing of antibiotic therapy is of great importance in order to achieve effective therapies by exceeding the minimum inhibitory concentrations of various pathogens, while also minimizing the risk of developing drug resistance. Our analysis revealed significant heterogeneity in dosing schemes, with a total of 11 different dosing schemes being identified. An initial dose of 1500 mg plus a dose(s) of 1500 mg administered at two-week interval was the most commonly used regimen, in 35.3% of cases, while the median duration of dalbavancin therapy was 2 weeks. Due to the high heterogeneity not only across different studies, but also among patients of single small case series, it is very difficult to extract concrete conclusions regarding the ideal dosing regimens. This is expected since there are not widely accepted recommendations guiding the optimal dosing of dalbavancin in patients with infective endocarditis. Interestingly, our findings indicate that a period of effective antimicrobial exposure of 2 weeks had similar success rate compared to those with a longer effective antimicrobial exposure. However, this finding should be interpreted with caution, since duration of treatment was not investigated based on disease severity or in special populations, thus a prolonged therapy of more than 2 weeks may be necessary in many cases. An additional concern relates to the potential development of antimicrobial resistance following prolonged or off-label suppressive use of dalbavancin, highlighting the need for prudent antibiotic stewardship. Further high-quality studies are needed to investigate the ideal duration of treatment for IE, based on the overall condition of patients, and the disease severity.

Another recent systematic review on dalbavancin effectiveness as a sequential therapy in patients with IE has been published by Leanza et al. [51]. The authors of this review focused on studies with more than 15 infective endocarditis cases, excluding small cases series, in order to minimize any small-study effect in the published literature. However, this methodological choice can also limit the number of included studies, resulting in a smaller overall population. Indeed, while our review included 565 cases of IE from 38 studies, the study by Leanza et al. analyzed 300 patients from 9 studies. Moreover, the authors of this study did not synthesize the cure rates from the various studies in order to provide aggregated outcomes. Nevertheless, both reviews consistently reported favorable outcomes with dalbavancin use, supporting its role as a treatment strategy in patients with infective endocarditis.

Turner et al. conducted the first randomized control trial (DOTS trial) evaluating the efficacy and safety of dalbavancin versus standard therapy for complicated *Staphylococcus aureus* bacteremia, including patients with right-sided endocarditis [60]. Following an initial course of 3–10 days of intravenous therapy, patients were allocated to receive either two doses of dalbavancin (1500 mg on days 1 and 8), or 4–8 total weeks of standard intravenous therapy (cefazolin, anti-staphylococcal penicillin, vancomycin or daptomycin). Dalbavancin met criteria for non-inferiority with respect to clinical efficacy but did not demonstrate superiority by desirability of outcome ranking at day 70. Although the results of this study support the use of dalbavancin as consolidation therapy in selected cases of complicated bacteremia and right-sided endocarditis, its findings cannot be directly extrapolated to the broader spectrum of infective endocarditis populations included in the present review, since patients with left-sided endocarditis, prosthetic valve endocarditis, and retained infected prosthetic material were excluded from the DOTS trial. The DOTS trial complements the existing observational evidence, highlighting the need for further randomized controlled trials targeting left-sided and prosthetic valve endocarditis.

There are several limitations regarding the external validity of our results, referring to the extent to which our findings can be generalized beyond the specific study context. First, there is a lack of clinical trials in this field while the evidence is grounded only on observational studies, with the majority consisting of retrospective studies, case series and case reports. Furthermore, there are no control groups in these observational studies, making it difficult to conduct a direct comparison with standard of care therapies. In addition, there are only a few studies focusing exclusively on infective endocarditis, thus there is no detailed information regarding several parameters such as the type of infective endocarditis and the disease severity. Another limitation is that the evaluation of the outcome was not consistent across the different studies, leading to a diverse definition of therapy failure. Therefore, some authors referred to the disease’s resolution as the remission of signs and symptoms, while others as the sterilization of blood cultures or the normalization of the laboratory values. In addition, endocarditis is a disease manifesting very often relapses, even months after treatment. However, the follow-up period was not standardized either, resulting in many cases with short follow-up periods that may be associated with false positive outcomes. Moreover, publication bias is inevitable concerning the high amount of case reports and assuming author’s preference to publish cases with positive outcomes. We also have to note that dalbavancin is mainly used as a consolidation therapy in patients with infective endocarditis that have already received previous antibiotics and have negative blood cultures. Information on antecedent antibiotic therapy before dalbavancin was highly heterogeneous and frequently incomplete across studies. Since administration of antibiotics prior (or concurrently) to dalbavancin may have a significant impact on the cure rate, it is impossible to extrapolate safe conclusions regarding the independent role of dalbavancin. Future studies should systematically report on the type and duration of previous antibiotic therapy prior to dalbavancin, in order to better define its contribution in sequential treatment regimens for infective endocarditis. Finally, a lack of consistent reporting of valve laterality (right-sided versus left-sided endocarditis) represents an important gap in the existing literature, limiting the ability to explore potential differences in outcomes according to disease location.

Based on the results of this systematic review, dalbavancin can be an efficient and safe antibiotic that may limit the burden of this devastating infection, with a cure rate of 88.0%. However, the role of dalbavancin needs to be investigated by randomized clinical trials that will allow us to identify the optimal dosing regimen and validate the effectiveness of dalbavancin as monotherapy. The findings of these clinical trials will guide future clinical practice recommendations, while they will also provide enough evidence to include infective endocarditis as an approved indication for dalbavancin.

## Supplementary Information

Below is the link to the electronic supplementary material.

Supplementary Material 1

## Data Availability

The datasets analyzed during the current study are available upon request to the authors of this study.

## Declarations

The authors declare that no funds, grants, or other support were received during the preparation of this manuscript.

All authors contributed to the study conception and design. Material preparation was performed by Anastasios E Chaldoupis, Andreas G Tsantes, Aglaia Domouchtsidou, Stella Baliou, Marianna Vlachaki, Alexandra Mpakosi, Sotirios P Fortis and George Samonis. Data collection was performed by Anastasios E Chaldoupis, Andreas G Tsantes, Vasilios Petrakis and Rozeta Sokou. Analysis was performed by Andreas G Tsantes, Gerasimos Siasos, Stefanos Bonovas, Petros Ioannou, Konstantina A Tsante, Deny Tsakri. The first draft of the manuscript was written by Anastasios E. Chaldoupis, Andreas G Tsantes and all authors commented on previous versions of the manuscript. All authors read and approved the final manuscript.

The datasets analyzed during the current study are available upon request to the authors of this study.
