# Supplementary material for: Dalbavancin for infective endocarditis: A systematic review of effectiveness, safety, and dosing
Source: Eur J Clin Microbiol Infect Dis. 2026 Feb 10;45(4):931–41. doi: 10.1007/s10096-026-05434-3 (PMC13086878; doi:10.1007/s10096-026-05434-3)
Supplement: Supplementary file 1 — Supplementary Material 1 [file 10096_2026_5434_MOESM1_ESM.docx]

Supplementary Table 1. Detailed search strategy

| PubMed and Scopus syntax | (New glycopeptides OR Lipoglycopeptides OR Telavancin OR Oritavancin OR Dalbavancin) AND Endocarditis |
| --- | --- |
